# Mechano-Sensing Channel PIEZO2 Enhances Invasive Phenotype in Triple-Negative Breast Cancer

**DOI:** 10.3390/ijms23179909

**Published:** 2022-08-31

**Authors:** Eriko Katsuta, Kazuaki Takabe, Marija Vujcic, Philip A. Gottlieb, Tao Dai, Arnaldo Mercado-Perez, Arthur Beyder, Qingfei Wang, Mateusz Opyrchal

**Affiliations:** 1 Department of Surgical Oncology, Roswell Park Comprehensive Cancer Center, Buffalo, NY 14263, USA; 2 Department of Surgery, University at Buffalo Jacobs School of Medicine and Biomedical Sciences, The State University of New York, Buffalo, NY 14203, USA; 3 Department of Breast Surgery and Oncology, Tokyo Medical University, Tokyo 160-8402, Japan; 4 Department of Surgery, Yokohama City University, Yokohama 236-0004, Japan; 5 Department of Surgery, Niigata University Graduate School of Medical and Dental Sciences, Niigata 951-8510, Japan; 6 Department of Breast Surgery, Fukushima Medical University, Fukushima 960-1295, Japan; 7 Department of Medicine, Roswell Park Comprehensive Cancer Center, Buffalo, NY 14263, USA; 8 Physiology and Biophysics, State University of New York at Buffalo, Buffalo, NY 14203, USA; 9 Department of Cell Stress Biology, Roswell Park Comprehensive Cancer Center, Buffalo, NY 14263, USA; 10 Enteric Neuroscience Program, Division of Gastroenterology & Hepatology, Department of Physiology and Biomedical Engineering, Mayo Clinic, Rochester, MN 55905, USA; 11 Department of Medicine, Division of Hematology/Oncology, Indiana University School of Medicine, Indianapolis, IN 46202, USA

**Keywords:** triple-negative breast cancer, PIEZO, mechano-signaling, metastasis

## Abstract

Background: Mechanically gated PIEZO channels lead to an influx of cations, activation of additional Ca^2+^ channels, and cell depolarization. This study aimed to investigate PIEZO2’s role in breast cancer. Methods: The clinical relevance of *PIEZO2* expression in breast cancer patient was analyzed in a publicly available dataset. Utilizing *PIEZO2* overexpressed breast cancer cells, and *in vitro* and *in vivo* experiments were conducted. Results: High expression of *PIEZO2* was correlated with a worse survival in triple-negative breast cancer (TNBC) but not in other subtypes. Increased PEIZO2 channel function was confirmed in *PIEZO2* overexpressed cells after mechanical stimulation. *PIEZO2* overexpressed cells showed increased motility and invasive phenotypes as well as higher expression of SNAIL and Vimentin and lower expression of E-cadherin in TNBC cells. Correspondingly, high expression of *PIEZO2* was correlated with the increased expression of epithelial–mesenchymal transition (EMT)-related genes in a TNBC patient. Activated Akt signaling was observed in *PIEZO2* overexpressed TNBC cells. *PIEZO2* overexpressed MDA-MB-231 cells formed a significantly higher number of lung metastases after orthotopic implantation. Conclusion: PIEZO2 activation led to enhanced SNAIL stabilization through Akt activation. It enhanced Vimentin and repressed E-cadherin transcription, resulting in increased metastatic potential and poor clinical outcomes in TNBC patients.

## 1. Introduction

Mechanical force is prevalent in our environment, and living organisms are able to detect it and respond to such a stimulus [1,2,3]. There is increasing evidence that cancer cells detect and respond to outside mechanical forces differently than non-transformed cells [4,5]. Furthermore, tumors have different physical properties than normal body tissues, leading to different mechano-signaling and cellular behavior [6]. Ion channels are essential for normal cellular function, highlighted by tight control of ion concentrations across the cytoplasmic membrane and within the cell. Dysregulation of ion channel function has been shown to be associated with many diseases such as cystic fibrosis [7], cardiac arrhythmias [8], epilepsy [9], renal disorders [10,11,12], and many others. Ion channels play an important role in malignancy, by interacting with oncogenic pathways [13,14], contributing to malignant transformation [15,16,17], and enhancing metastasis [17,18]. Many of the oldest and most successful drugs have targeted ion channels [19].

Mechanically gated PIEZO ion channels play an important role in mechano-sensing and mechano-transduction [7,20,21]. PIEZO, a family of mechanically gated channels, is being actively studied in connection with cellular functions and cancer. PIEZO1 channel signaling has been shown to increase the motility and migration of breast cancer cells [22]. PIEZO1 has been also shown to be correlated with increased migration and chemo-resistance in gastric cancer cells [23], increased invasiveness of osteosarcoma cells [24], and increased cell viability in synovial sarcoma cells [25]. On the contrary, a lower level of PIEZO1 is correlated with an invasive phenotype in lung cancer [26,27]. Mechanical activation of PIEZO2 leads to an influx of cations and activates additional Ca^2+^ channels, resulting in cell depolarization [28]. There is early evidence that PIEZO2 may also be involved in carcinogenesis and cancer progression [27,29]. PIEZO2-mediated Ca^2+^ influx regulates cytoskeleton through RhoA activity [30], both of which are important for cellular motility [31].

The mechano-signaling in cancer remains poorly understood and its importance in tumorigenesis and progression remains unknown. Early evidence of mechanical signaling comes from the observation that cancer cells are stiffer than the surrounding tissue [32]. The tumor microenvironment continuously exerts mechanical forces on the tumor cells, as a result of increased tumor mass, increased interstitial pressure from fluid extravasation due to leaky vasculature and lymphatic dysfunction, and changes in the composition of the surrounding matrix. In breast cancer, the connection between breast density and increased incidence risk has been described [33,34]. Therefore, identifying the specific components of the signaling pathways regulating cancer cell response to mechanical stress will deepen our understanding of the disease and hopefully lead to the discovery of novel targets.

We hypothesized that PIEZO2 plays a significant role in breast cancer biology. In this study, we investigated PIEZO2’s roles in the breast cancer phenotype, using *in vitro* and *in vivo* experiments together with a bioinformatical approach.

## 2. Results

### 2.1. Increased PIEZO2 Expression Is Associated with Poor Prognosis in TNBC Patients

**Figure 1:**
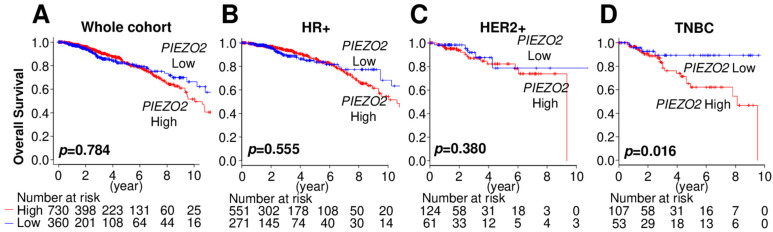
Increased *PIEZO2* expression is associated with poor clinical outcomes in patients with TNBC. (**A**) Breast cancer prognosis by *PIEZO2* expression in the whole cohort, (**B**) hormone receptor-positive (HR+), (**C**) HER2-positive (HER2+), and (**D**) triple-negative breast cancer (TNBC) of TCGA cohort. Red line represents *PIEZO2* high tumors and blue line represent *PIEZO2* low tumors.

We first investigated the impact of *PIEZO2* mRNA expression level on breast cancer patients’ prognosis using a TCGA cohort. *PIEZO2* expression was not correlated with overall survival in whole breast cancer patients (Figure 1A). Similarly, there were no survival differences between the *PIEZO2* high and low groups in the hormone receptor (HR)-positive cohort (Figure 1B) or HER2-positive cohort (Figure 1C). Notably, patients with higher *PIEZO2* expression tumors showed significantly worse overall survival rates compared to that with lower expression (*p* = 0.016) in the TNBC cohort (Figure 1D). Therefore, we focused on investigating the role of PIEZO2 in TNBC cell biology.

### 2.2. PIEZO2 Promotes Invasion and Migration of TNBC Cells

**Figure 2:**
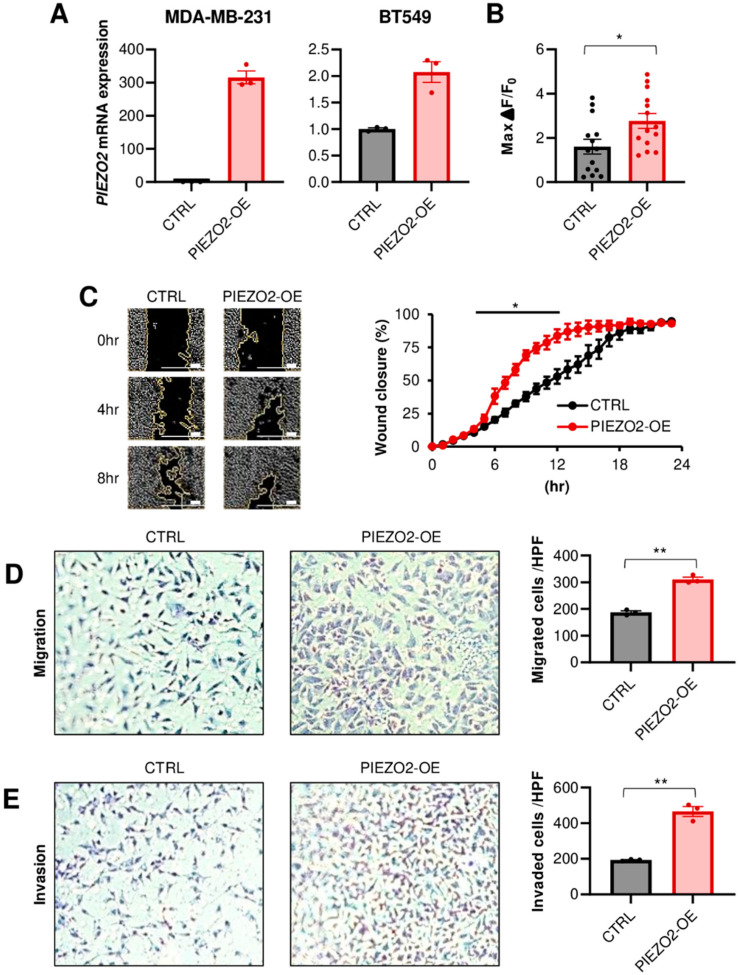
Overexpression of PIEZO2 leads to invasive phenotype *in vitro*. (**A**) *PIEZO2* mRNA measured by qPCR in control (CTRL) or *PIEZO2* inserted vector (PIEZO2-OE) transfected MDA-MB-231 and BT549 cells. (**B**) Changes in intracellular Ca^2+^ after mechanical stimulation in control or *PIEZO2* overexpressed MDA-MB-231 cells. *n* = 13, each. (**C**) Wound healing assay of control or *PIEZO2* overexpressed MDA-MB-231 cells. The scale bar indicates 100 µm. Transwell migration assay (**D**) and invasion assay (**E**) in control and *PIEZO2* overexpressed MDA-MB-231 cells. * *p* < 0.05, ** *p* < 0.01.

We next investigated whether PIEZO2 affects the phenotype in TNBC cells. To this end, MDA-MB-231 and BT549 cell lines were used, since they have intermediate *PIEZO2* expression levels, measured by qPCR, among eight TNBC cell lines (Figure S1). We first stably overexpressed *PIEZO2* in these two cell lines. The overexpression was verified by qPCR, as shown in Figure 2A. Next, we assessed if this ectopic overexpressed *PIEZO2* could serve as a functioning ion channel. We observed that *PIEZO2* overexpression leads to an increased intracellular Ca^2+^ current, after mechanical stimulation with a probe in MDA-MB-231, demonstrating a functional change in mechano-sensing (Figure 2B and Figure S2). Although *PIEZO2* overexpression did not change cell proliferation (Figure S3), *PIEZO2* overexpressing cells showed higher migration capability in both a wound healing assay (*p* < 0.05) (Figure 2C) and a double chamber migration assay (*p* < 0.01) (Figure 2D). *PIEZO2* overexpressing MDA-MB-231 cells showed a significantly increased ability to invade through a membrane, compared to the control cells, in the double chamber invasion assay (*p* < 0.01) (Figure 2E). These findings suggest that increased PIEZO2 promotes the migration and invasion capability of TNBC cells.

### 2.3. PIEZO2 Overexpression Leads to Upregulation of Genes Associated with Aggressive TNBC Phenotype

**Figure 3:**
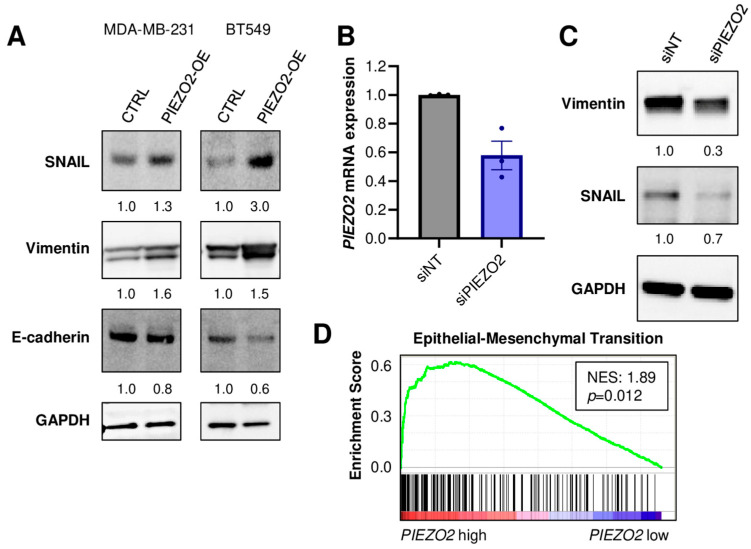
PIEZO2 level leads to expression changes of genes associated with invasion and metastasis in TNBC. (**A**) Western blotting of SNAIL, Vimentin, E-cadherin, and GAPDH in control (CTRL) and *PIEZO2* overexpressed (PIEZO2-OE) MDA-MB-231 and BT549 cells. (**B**) *PIEZO2* mRNA quantified by qPCR in siRNA non-targeted (siNT) or for *PIEZO2* (siPIEZO2)-transfected MDA-MB-231 cells. (**C**) Western blot of Vimentin, SNAIL, and GAPDH in control (siNT) and *PIEZO2* knockdown (siPIEZO2) MDA-MB-231 cells. (**D**) Gene-set enrichment analysis comparing *PIEZO2* high- and low-expressing TNBCs in TCGA cohort.

We investigated the mechanisms of *PIEZO2* overexpression that induced invasive phenotypes. We found that increased expressions of SNAIL and Vimentin in *PIEZO2* overexpressed MDA-MB-231 and BT549 cells. E-cadherin expression was decreased in *PIEZO2* overexpressed BT549 cells, whereas a very minor reduction was seen in MDA-MB-231 cells (Figure 3A). We next explored the effect of PIEZO2 downregulation on these proteins. Knockdown of *PIEZO2* by siRNA, as confirmed by qPCR (Figure 3B), led to a decrease in expression of both Vimentin and SNAIL (Figure 3C). Furthermore, patient transcriptomic analysis supported these results, where 103 out of 195 epithelial–mesenchymal transition (EMT)-related genes were upregulated in *PIEZO2* high-expression tumors, compared to low-expression tumors in the TCGA–TNBC cohort (NES 1.89, *p* = 0.012) (Figure 3D). These findings suggest that increased PIEZO2 expression leads to expression changes of the genes related to increased aggressiveness and poorer prognosis in TNBC.

### 2.4. Overexpression of PIEZO2 Has No Significant Impact on YAP/TAZ Signaling Pathway

**Figure 4:**
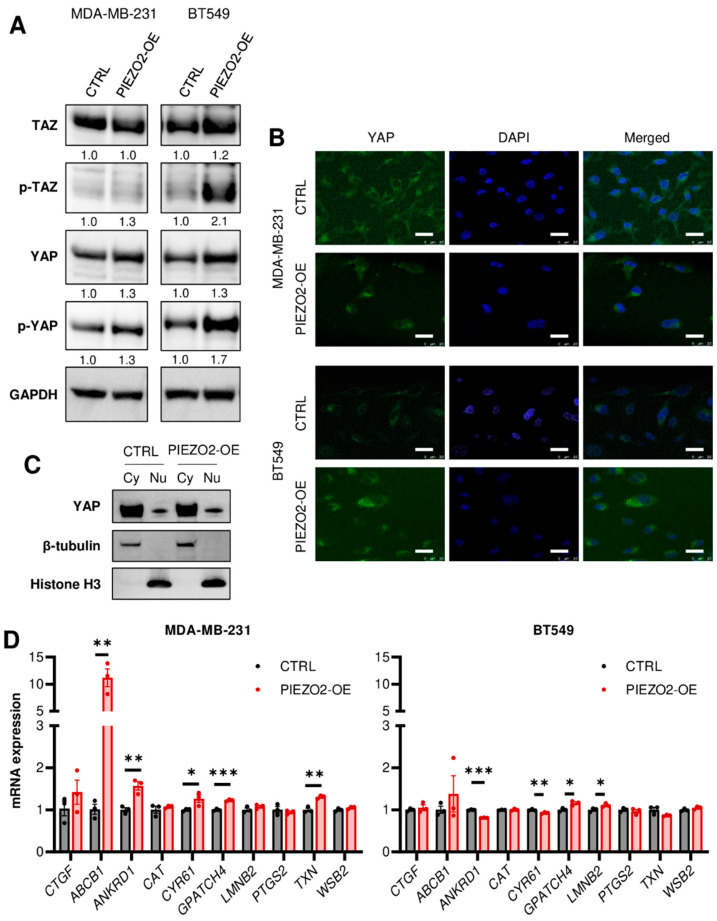
Overexpression of PIEZO2 has no significant impact on YAP/TAZ signaling pathway. (**A**) Western blotting of TAZ, p-TAZ, YAP, p-YAP, and GAPDH in control (CTRL) and *PIEZO2* overexpressed (PIEZO2-OE) MDA-MB-231 and BT549 cells. (**B**) Representative immunofluorescent images of YAP with nuclear DAPI staining in control and *PIEZO2* overexpressed cells in MDA-MB-231 and BT549. Scale bar indicates 25 µm. (**C**) Western blot of YAP, β-tubulin, Histon H3 in the cytosol (Cy), and nuclear (Nu) fraction of MDA-MB-231 control and PIEZO2 overexpressed cells. (**D**) YAP/TAZ target genes expressions in control and *PIEZO2* overexpressed cells in MDA-MB-231 and BT549. * *p* < 0.05, ** *p* < 0.01, and *** *p* < 0.001.

YAP/TAZ has been shown to be regulated through mechano-signaling [35,36,37,38,39] and SNAIL [40,41]. YAP nuclear translocation leads to increased gene transcription and results in a more aggressive cancer phenotype. Nuclear translocation is inhibited by YAP/TAZ phosphorylation [42]. Thus, we hypothesized that PIEZO2 promotes invasion and migration through YAP nuclear translocation. We observed increased phosphorylation of YAP in both MDA-MB-231 and BT549 cells with overexpressed *PIEZO2* together with slightly increased total YAP (Figure 4A). Overall, TAZ levels showed no apparent change in MDA-MB-231 cells with an increase in phosphorylated TAZ in BT549 PIEZO2 overexpressed cells (Figure 4A). We further evaluated changes in YAP nuclear translocation with *PIEZO2* overexpression. YAP nuclear translocation was rarely seen by immunofluorescent staining (<1%), regardless of *PIEZO2* expression level in both MDA-MB-231 and BT549 (Figure 4B and Figure S4). This finding was further confirmed by Western blotting, where YAP level in the nuclear fraction of the *PIEZO2* overexpressed cells was similar to that of the control in MDA-MB-231 cells (Figure 4C). We also investigated changes in YAP/TAZ target gene expressions [39]. *ABCB1* mRNA was higher in the *PIEZO2* overexpressed cells as compared to the control, and *ANKRD1*, *CYP61*, *GPATCH4*, and *TXN* also had a small but statistically significant increase in MDA-MB-231 cells. Among those five genes, only *GPATCH4* showed statistically significant increase in BT549 cells, with the rest showing no change or a decrease (Figure 4D). Therefore, we did not see any conclusive evidence that PIEZO2 regulates YAP/TAZ signaling in MDA-MB-231 and BT549 breast cancer cells.

### 2.5. PIEZO2 Overexpression Results in Activation Akt/GSK-3β Signaling Pathway

**Figure 5:**
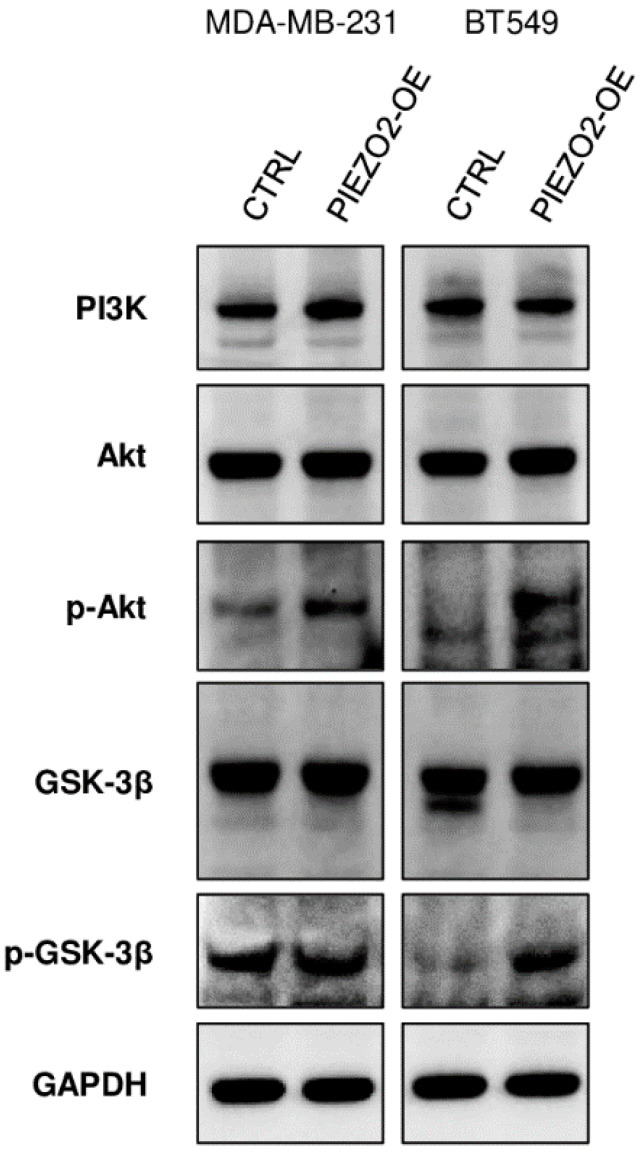
Overexpression of *PIEZO2* showed activation of Akt/GSK-3β pathway. Western blotting of PI3K, Akt, p-Akt, GSK-3β, p-GSK-3β, and GAPDH in control (CTRL), and *PIEZO2* overexpressed (PIEZO2-OE) in MDA-MB-231 and BT549 cells.

We further investigated the mechanism of how PIEZO2 overexpression results in increased SNAIL. SNAIL stability and nuclear translocation is inhibited by GSK-3β, which phosphorylates SNAIL leading to its degradation [43]. GSK-3β is regulated by PI3K/Akt pathway where activated Akt inactivates GSK-3β by phosphorylating it at Ser9. [44,45]. As shown in Figure 5, *PIEZO2* overexpressed cells showed enhanced phosphorylated Akt in both MDA-MB-231 and BT549 cells compared to the controls. Further, we observed an increase in phosphorylated GSK-3β in *PIEZO2* overexpressed BT549 cells, whereas the change was minor in MDA-MB-231. Overall, PI3K level was unchanged in both MDA-MB-231 and BT549 cells. These results suggest that PIEZO2 may drive Akt activation, resulting in increased stability and nuclear translocation of SNAIL, leading to an increased metastatic phenotype.

### 2.6. PIEZO2 Overexpression Promotes Lung Metastasis

**Figure 6:**
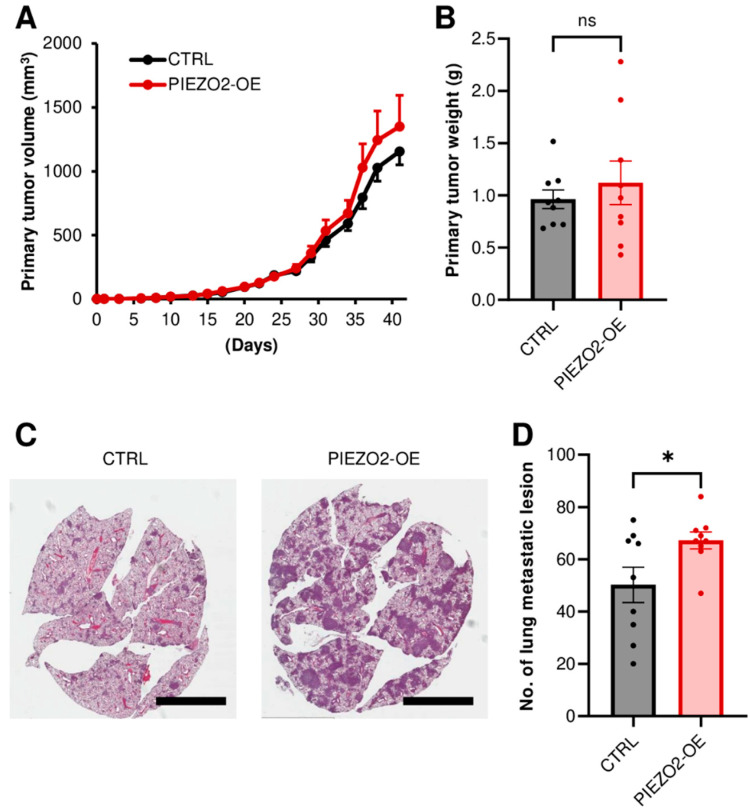
PIEZO2 upregulation increases lung metastasis of TNBC cells. (**A**) Tumor growth curves of control (CTRL) and *PIEZO2* overexpressed (PIEZO2-OE) MDA-MB-231 in mice. (**B**) Comparison of primary tumor weight between control and *PIEZO2* overexpressed tumors at the endpoint. (**C**) Representative images of lung metastasis. Scale bar indicates 5 mm. (**D**) Comparison of lung macrometastatic lesion numbers in control and PIEZO2 overexpressed groups. *n* = 9, each. * *p* < 0.05.

We further investigated the metastatic capability in the *PIEZO2* overexpression using MDA-MB-231 cells in a mouse xenograft model. Either the control or *PIEZO2* overexpressed cells were implanted into mouse mammary fat pads. To fully evaluate metastasis, the experiment was terminated before the size difference was seen in the primary tumors (Figure 6A). We confirmed the equity of the primary tumor growth between the two groups, by comparing the resected tumor weight. There was no statistical difference in tumor weight, although the PIEZO2 overexpressed group trended to be slightly heavier (Figure 6B). Next, we examined the lung macrometastasis by counting the visible tumors in the whole lung sections. There was a significant increase in the number of lung macro-metastatic nodules in the *PIEZO2* overexpressed group, as compared to the controls (*p* < 0.05) (Figure 6C,D), and the metastases were also qualitatively larger in most sections, as seen in the representative images (Figure 6C), suggesting that *PIEZO2* overexpressed MDA-MB-231 cells have a higher capability to form lung metastasis.

### 2.7. Proposed Model for PIEZO2-Induced Invasive Phenotype

**Figure 7:**
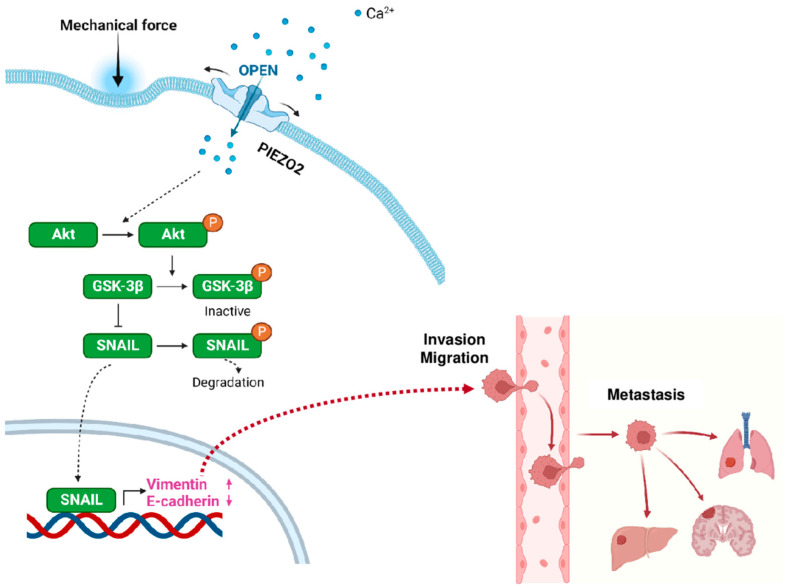
Proposed model for PIEZO2 and its role in metastatic phenotype of triple-negative breast cancer.

Based on the experimental results, we propose a model of how PIEZO2 leads to invasive phenotype in TNBC. We propose that the external mechanical force activates PIEZO2, resulting in an inflow of cations (Ca^2+^ among them), which leads to Akt activation by its phosphorylation. Activated Akt inactivates GSK-3β by phosphorylating its Ser9 residue, leading to SNAIL stability and its nuclear translocation (Figure 7). SNAIL has been shown to induce mesenchymal phenotype [46]. Activated SNAIL enhances Vimentin and represses E-cadherin transcriptions [47], resulting in a more invasive phenotype with increased capability to form metastases, which are shown to correlate in worse clinical outcomes in TNBC patients. These series of events are independent of YAP signaling.

## 3. Discussion

In this study, we found that high expression of *PIEZO2* was correlated with a worse prognosis in TNBC. We showed an increased Ca^2+^ current, in response to the mechanical force in *PIEZO2* overexpressed cells, demonstrating that the functioning ion channel was increased in *PIEZO2* overexpressed TNBC cells. Higher expressions of SNAIL and Vimentin and lower expression of E-cadherin were observed in *PIEZO2* overexpressed cells, as compared to the control in TNBC cells. These findings were supported by the gene-expression profile in patient samples, where a high *PIEZO2* level was correlated with higher expression of the genes associated with EMT in the TCGA–TNBC cohort. Increased YAP nuclear translocation has been demonstrated with increased mechanical forces in the previous studies [35]; however, we did not see solid evidence that PIEZO2 induced YAP nuclear translocation. On the other hand, we observed an activated Akt/GSK-3β pathway in *PIEZO2* overexpressing cells. Therefore, we propose a model, where PIEZO2 activity leads to Akt pathway activation and increased SNAIL stability and nuclear translocation, resulting in the enhancement of Vimentin and repressing of E-cadherin transcription in TNBC. It, in turn, leads to increased cell motility and invasiveness, which eventually promotes metastasis.

PIEZO2 function has been best described in neuronal tissues [48]. It has been shown to be essential in sensory processes [49,50,51,52,53,54]. The role of PIEZO2 in cancer has not been elucidated, although there is evidence of its involvement with angiogenesis [29]. PIEZO2 has been proposed to play a role in embryonic development [55], cell migration [56] and cell differentiation [57], which are all important in carcinogenesis and cancer progression. We demonstrated that PIEZO2 expression levels are inversely correlated with clinical outcomes in only TNBC. Different cancer types may sense mechanical forces through different means. Many channels that have been identified to at least partially respond to mechanical force [58,59]. PIEZO2’s role in non-TNBC, as well as in other types of cancer, is yet to be determined. Further studies are needed to fully elucidate PIEZO2 roles in cancer.

We sought to identify the mechanisms of how PIEZO2’s signal activates SNAIL. There is evidence that mechanical signal transduction leads to cancer aggressiveness through YAP nuclear translocation, which is inhibited by YAP/TAZ phosphorylation [35,36,37,38,39]. Therefore, we hypothesized that the PIEZO2 signal promotes YAP nuclear translocation. However, we observed increased phosphorylated YAP and TAZ. Further, we did not see the increased YAP nuclear translocation in *PIEZO2* overexpressed cells. We also observed only one obvious gene upregulation among 10 examined YAP target-gene expressions in *PIEZO2* overexpressed MDA-MB-231 cells and very minor changes in BT549 cells. These results highlight the complex nature of the interactions.

We demonstrated that *PIEZO2* overexpressing cells showed enhanced Akt phosphorylation. The previous reports support our findings that mechanical force induces Akt activation in endothelium [60], and mechano-signaling activates the PI3K/Akt pathway in breast cancer [61]. Further, it has been shown that activated Akt leads to SNAIL protein stabilization and nuclear translocation [44,45,62]. Therefore, we proposed a model where PEIZO2 activates the Akt pathway, leading to enhanced SNAIL transcription factor activity.

Although we proposed a model, in which increased cations through PIEZO2 lead to upregulation of SNAIL by Akt signaling, which further enhances Vimentin and represses E-cadherin transcription, resulting in a more invasive phenotype in TNBC, there are still gaps in the understanding of how PIEZO2 results in increased metastatic phenotype. Previously, it was reported that PIEZO2 mechano-signaling regulates RhoA and the cytoskeleton to promote migration and extracellular matrix degradation in brain metastatic TNBC cells [30]. RhoA and its downstream ROCK regulate the cytoskeleton, which can promote cell migration through its effects on integrins and cell adhesion [63,64]. Therefore, it may be involved in PIEZO2/SNAIL connections. The link between PIEZO2 RhoA/ROCK pathways and details of the interaction with Akt need to be explored.

Our work shows that PIEZO2 is involved in aggressive phenotypes, which correlates with clinical outcomes in TNBC. Further studies of the roles of PIEZO2 channel and mechanical signaling are needed to better understand the full scope in TNBC.

## 4. Materials and Methods

### 4.1. Bioinformatic Analyses

Gene expression from RNA-sequence and clinical data of TCGA cohort were downloaded through cBioportal (https://www.cbioportal.org/, TCGA provisional dataset, downloaded on 21 June 2018) [65,66]. Patients were divided into *PIEZO2* high and low groups using a lower tertile cutoff.

Gene Set Enrichment Analysis (GSEA) was carried out, comparing transcriptomic profiles between *PIEZO2* high and low expression tumors in TCGA TNBC patients using software provided by the Broad Institute (https://www.gsea-msigdb.org/gsea/index.jsp, downloaded on 21 June 2018).

### 4.2. Cell Culture and Reagents

Human TNBC cell lines, MDA-MB-231, and BT549 were obtained from ATCC (Manassas, VA, USA), and cultured in Dulbecco’s Modified Eagle Medium (DMEM) (Gibco, Gaitherburg, MD, USA) with 10% fetal bovine serum (FBS) (Gibco). All cell lines were used in the present experiments within 20 passages after the reception. All cell lines were routinely tested to rule out mycoplasma infection using PlasmoTest kit (InvivoGen, San Diego, CA, USA). All cell lines were cultivated in a humidified incubator at 37 °C in 5% CO_2_.

Human *PIEZO2* specific siRNA and non-targeting siRNA (Dharmacon, Lafayette, CO, USA) were transfected into the MDA-MB-231 using lipofectamine RNAiMAX, in accordance with the instructions of the manufacturer. The siRNA-treated cells were collected 48 h after transfection. *PIEZO2* expression was determined by qPCR, and cells were utilized for further experiments. Either empty or *PIEZO2*-inserted pcDNA3.1 was transfected to MDA-MB-231 and BT549 cells utilizing jetPRIME (Polyplus transfection, Illkirch, France). The cells were selected by G418 treatment to generate stably PIEZO2 overexpressed cells. *PIEZO2* level was confirmed by qPCR.

### 4.3. qPCR

Total RNA was extracted utilizing RNeasy Mini Kit (Qiagen, Hilden, Germany), and cDNA was synthesized utilizing High-Capacity cDNA Reverse Transcription Kit (Applied Biosystems, Foster City, CA, USA), as described before [67]. Primer sequences were listed in Table S1. Data were analyzed using the 2^ΔΔCt^ method. *GAPDH* was used as the internal control.

### 4.4. Calcium Current Measurement

The control or *PIEZO2* overexpressed MDA-MB-231 cells were co-transfected with GCamP5 (CMV-GCaMP5G: Addgene #31788) and tdTomato (tdTomato-C1: Addgene #54653) plasmids using the Lipofectamine 3000 (ThermoFisher Scientific, Waltham, MA). Calcium current was measured, as previously reported [68,69] and briefly explained below. Bath solution contained: 127 mM NaCl, 3 mM KCl, 1 mM MgCl_2_·6H_2_O, 2.5 mM CaCl_2_·2H_2_O, 10 mM glucose, and 10 mM HEPES, pH 7.35. Transfected cells were identified on an inverted epifluorescence microscope (Olympus IX70) via RFP fluorescence. Functional studies were done visualizing GCamP5. pCLAMP 10.6 software (Molecular Devices, San Jose, CA, USA) was used to drive the concerted operation of a 16-bit high-speed camera (ORCA-Flash4.0, Hamamatsu), a LED illumination system (CoolLED pE-300Ultra, CoolLED Limited, Andover, UK), and MetaMorph Software (Molecular Devices) for acquisition. Images were captured at a frame rate of 5 Hz. Mechanical stimulation was carried out using an electric-driven fire-polished glass probe (1 µm indentation, 50 ms duration) driven by a transducer P-621.1CD with an E-625.CR controller (PI, Physik Instrument, Auburn, MA, USA), also controlled with the pCLAMP software. All experiments were performed at room temperature (25 °C). Analysis of imaging data was performed within MetaMorph by subtracting the background and then by analyzing peak responses in selected cells, compared to the pre-stimulation baseline, to calculate ΔF/F_0_.

### 4.5. Western Blotting

Cells were lysed with RIPA lysis buffer. The lysate was separated by electrophoresis and transferred to the PVDF membrane (BioRad, Hercules, CA, USA). The membrane was blocked with 5% milk for 1 h at room temperature, and then incubated with primary antibody at 4 °C overnight. The primary antibodies and their dilutions used in this study were listed in Table S2. Bands were developed with HRP-labelled secondary antibodies (anti-rabbit, BioRad) followed by Clarity Western ECL detection system (BioRad). Chemiluminescence signal was acquired using a ChemiDoc MP imager (BioRad).

### 4.6. Transwell Assay

Transwell assay was conducted for invasion and migration assay. Inserts with and without Matrigel were used for invasion and migration assay, respectively. Then, 1 × 10^5^ cells in 0.1 mL of serum-free media were placed on top of the transwell membrane with 8.0 µm pore, and 600 µL of the DMEM media with 10% FBS was placed in the lower chamber in the 24-well plate. After 16 h incubation, transwell membranes were stained with 0.1% crystal violet and then invaded, and migrated cells were counted in three separate areas of each membrane.

### 4.7. Wound Healing Assay

Cells were cultured in a 24-well plate with the 2 well insets (Ibidi, Martinsried, Germany). When the cell density reached confluent, the chamber was removed. The plate was put in the BioSpa 8 incubator (BioTek, Winooski, VT, USA), and images were captured every 1 h by Cytation 5 cell imaging (BioTek). Analysis of the images was handled by Gen5 Image Prime software (BioTek).

### 4.8. Cell Proliferation Assay

Then, 3000 cells were seeded per well in a 96-well plate. At indicating time point, viable cells were quantified using CCK-8 kit (Dojindo, Kumamoto, Japan), in accordance with the instructions of the manufacturer.

### 4.9. Nuclear YAP Staining

The cells were seeded and incubated on cover glass overnight. Cells were fixed with 10% trichloroacetic acid (Sigma, St. Louis, MO, USA) for 15 min at 4 °C, incubated in 0.2% Triton (Fisher)-PBS for permeabilization for 5 min at room temperature, and then incubated in 3% bovine serum albumin (Sigma)-PBS for blocking for 1 h at room temperature. The slides were further incubated with the fluorescent conjugated primary antibodies; YAP Alexa Flour 488 conjugated (1:100, Sant Cruz; sc-376830, Dallas, TX, USA) for another hour at room temperature. After the mounting using DAPI contained mounting medium (Duolink, Sigma), the images were captured by a confocal fluorescent microscope.

### 4.10. Animal Study

The animal study protocol was approved by Roswell Park Cancer Institution Animal Care and Use Committee. CB17 SCID mice (female, 6–8 weeks-old, 18–22 g) were purchased in-house from Roswell Park Comprehensive Cancer Center. A total of 1 × 10^6^ cells in 20 μL suspension (10% PBS and 90% Matrigel) was implanted into mouse chest mammary fat pads. Tumor growth was evaluated by caliper measurement every 2 or 3 days until endpoint. Tumor volumes were calculated as 0.5 × (length) × (width)^2^. At the endpoint, lungs were harvested and fixed with 10% formalin, embedded in paraffin block, and then sectioned at the maximum cross-section for further analysis. The 5 µm thick slides were stained with hematoxylin and eosin. The tumor nodules were examined under a microscope.

### 4.11. Statistical Analysis

Data were represented as the mean and standard error of mean. A continuous value between two groups was compared by Student’s t-test, and ANOVA with post hoc Tukey’s was used for comparison of more than two groups. The survival differences were analyzed using Kaplan–Meier curves with the log-rank test. All statistical analyses were performed using Prism version 9.1.0 (GraphPad, San Diego, CA, USA) and R software version 4.2.1 (http:///www.r-project.org/) together with Bioconductor version 3.15 (http://bioconductor.org/).

## Data Availability

A publicly available dataset was analyzed in this study. This data can be found here: https://www.cbioportal.org/.

## Supplementary Materials

The following supporting information can be downloaded at: https://www.mdpi.com/article/10.3390/ijms23179909/s1.
