# Supplementary material for: Mechano-Sensing Channel PIEZO2 Enhances Invasive Phenotype in Triple-Negative Breast Cancer
Source: Int J Mol Sci. 2022 Aug 31;23(17):9909. doi: 10.3390/ijms23179909 (PMC9455988; doi:10.3390/ijms23179909)
Supplement: Supplementary file 1 [file ijms-23-09909-s001.zip › ijms-1862134-supplementary.pdf]

International Journal of
Molecular Sciences

MDPI

Article

Supplementary Materials: Mechano-sensing channel PIEZO2 enhances invasive phenotype in triple-negative breast cancer

Eriko Katsuta [1], Kazuaki Takabe [1-6], Marija Vujcic [7], Philip A. Gottlieb [8], Tao Dai [9], Arnaldo Mercado-Perez [10], Arthur Beyder [10], Qingfei Wang [11], Mateusz Opyrchal [7,11,*]

Table S1. qPCR primer sequence.

Gene	F	R
PIEZO2	TGCACAGTCAAAGGCTATCAA	ATGCTGTCCCAAATGATTCC
CTGF	AGGAGTGGGTGTGTGACGA	CCAGGCAGTTGGCTCTAATC
ABCB1	TCACTATTGTTTCTAGCCCTTGG	ATGTATCGGAGCCGCTTG
ANKRD1	CGAATTCCGTGATATGCTTGA	AAACATCCAGGTTTCCTCCA
CAT	GGATCACATACTTTCAAGCTGGT	TTTTTGATGCCCTGGTCAGT
CYR61	ACCCTTCTCCACTTGACCAG	CTTGGCGCAGACCTTACAG
GPATCH4	CCAAGCCCAACTTGCTGTAT	TGCAGCTCTCCAAGTCTTTGT
LMNB2	CCCACTCCTCATCTCACCAT	GCCCAAGCATCTTCTAAACCT
PTGS2	TGGGAAGCCTTCTCTAACCTC	TCAGGAAGCTGCTTTTTACCTT
TXN	TTACAGCCGCTCGTCAGA	AAGGCTTCCTGAAAAGCAGTC
WSB2	TGTGATTATGTGGGACCCCTA	CAGATCTCAGTGAGCTAATGTGG
GAPDH	AAGGTGAAGGTCGGAGTCAAC	GGGGTCATTGATGGCAACAATA

Table S2. List of antibodies for western blot.

Primary antibodies	Company	species	type	dilution	reference
SNAIL	CST	rabbit	monoclonal	1:300	3879S
Vimentin	CST	rabbit	monoclonal	1:3,000	5741S
E-cadherin	CST	rabbit	monoclonal	1:1,000	3195S
YAP/TAZ	CST	rabbit	monoclonal	1:1,000	8418S
p-TAZ	CST	rabbit	monoclonal	1:300	59971S
p-YAP	CST	rabbit	monoclonal	1:1,000	13008S
PI3K	CST	rabbit	monoclonal	1:1,000	4249S
Akt	CST	rabbit	monoclonal	1:1,000	9272S
p-Akt	CST	rabbit	monoclonal	1:1,000	4060S
GSK-3β	CST	rabbit	monoclonal	1:1,000	12456S
p-GSK-3β	CST	rabbit	monoclonal	1:500	9336S
GAPDH	CST	rabbit	monoclonal	1:10,000	5174S
YAP (for nuclear fraction)	CST	rabbit	monoclonal	1:1,000	14074T
β-Tubulin	CST	rabbit	monoclonal	1:1,000	2128S
Histone H3	CST	rabbit	monoclonal	1:1,000	4499S

CST: Cell Signaling Technologies

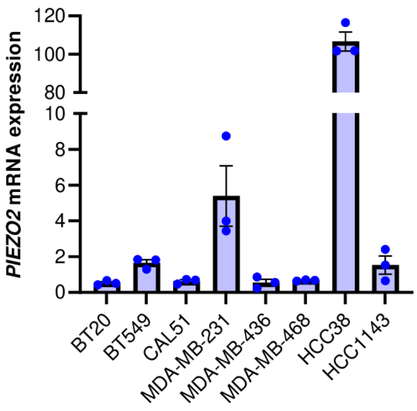

Figure S1. *PIEZO2* mRNA expression in triple-negative breast cancer (TNBC). *PIEZO2* mRNA expression in TNBC cell lines. Data was normalized with normal breast epithelial cell line, MCF10A as 1.

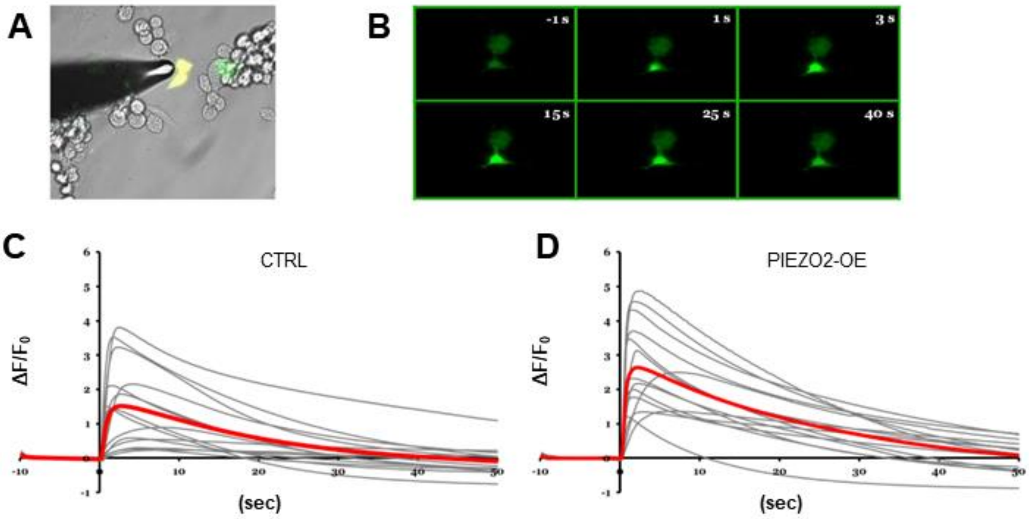

Figure S2. *PIEZO2* overexpression increases mechanically evoked calcium transients in MDA-MB-231 cells. MDA-MB-231 control (CTRL) or *PIEZO2* overexpressing cells (PIEZO2-OE) were transfected tdTomato for identification and GCamP5, a genetically encoded indicator of cytoplasmic Ca^{2+} concentration. (**A**) Overlaid difference interference contrast (DIC)/epifluorescence image showing cells co-transfected with the red tdTomato and the green GCamP5 (yellow). To the left, an electrically-driven blunted glass pipette is visualized. It delivers a 50 ms mechanical stimulus to the cells. Image magnification is 40x. (**B**) Stills from a green fluorescence time-lapse showing a representative experiment. The blunted pipette stimulates the cell at 0 sec. Typical fluorescence before mechanical stimulation (baseline, $t < 0$ s), followed by stimulation ($t > 0$ s), as the fluorescence sharply increases in response to the mechanical stimulus and returns to the pre-stimulation baseline. Image magnification is 40x. Normalized traces of GCaMP5 fluorescence for (**C**) CTRL and (**D**) PIEZO2-OE cells. Gray traces represent individual cells. Red traces represent the cumulative average. Negative time values are prior to mechanical stimulation. Positive time values are after mechanical stimulation. $\Delta F/F_0$ = difference in fluorescence from baseline normalized to baseline.

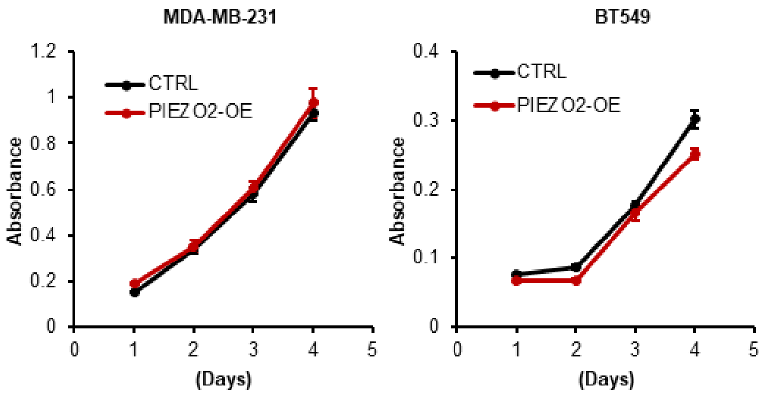

Figure S3. Proliferation of cells quantified by CCK8 in control (CTRL) and *PIEZO2* overexpressed (PIEZO2-OE) MDA-MB-231 and BT549 cells. *n*=6, each.

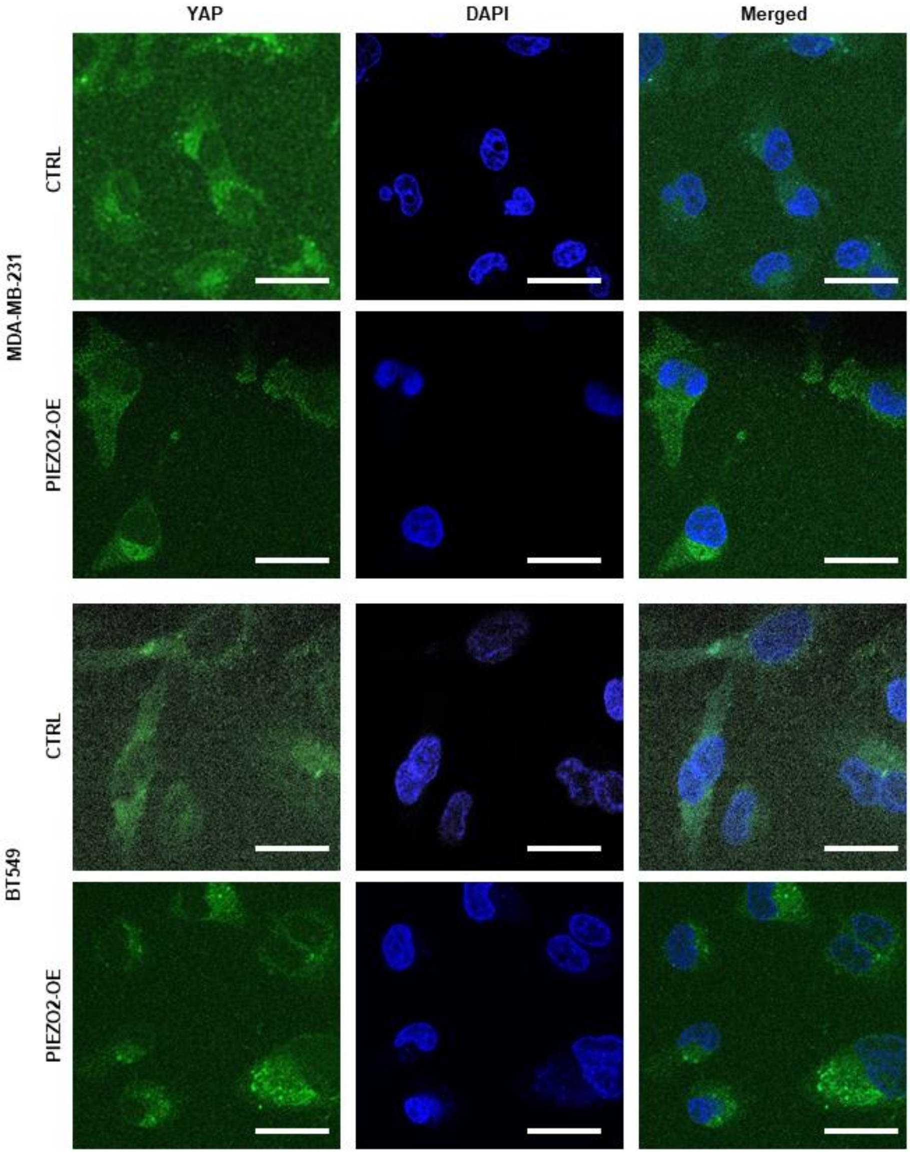

Figure S4. Representative images of YAP staining. YAP staining in control (CTRL) and *PIEZO2* overexpressed (PIEZO2-OE) cells of MDA-MB-231 and BT549. Scale bar indicates 25 μm.
